# Health-related factors of the Iraqi adult population during the 2020 COVID-19 pandemic: physical activity, eating behavior, quality of life, general health, and mood states cross-talk

**DOI:** 10.1186/s12889-023-15898-z

**Published:** 2023-06-01

**Authors:** Hiwa Ahmed Rahim, Rastegar Hoseini, Zahra Hoseini, Eman Najemaldeen Abbas, Dashni Anwer Kareem

**Affiliations:** 1 Physical Education and Sport Sciences Department, University of Halabja, Kurdistan Region, Halabja, 46018 Iraq; 2 Department of Exercise Physiology, Faculty of Sport Sciences, Razi University, P.O.Box. 6714414971, Kermanshah, Iran; 3 Physical Education and Sport Sciences Department, University of Sulaimani, Kurdistan, Iraq

**Keywords:** Exercise, Lockdown, Coronavirus, Health

## Abstract

**Background:**

The lockdown and social distancing caused by Coronavirus disease 2019 (COVID-19) may have changed Physical Activity Level (PAL), eating behavior, and health habits due to long-term confinement worldwide.

**Objective:**

This study aimed to evaluate the PAL, eating behavior, Quality of Life (QoL), General Health (GH), and mood states during COVID-19 confinement in a large sample of Iraqi adults.

**Methods:**

3738 healthy adults (age 18–70 years) residing in Halabjeh, Iraq answered the online questionnaires including the short form of international physical activity, GH, three-factor eating (TFEQ-R18), and a short form of the profile of mood states (POMS-SF) questionnaires. Data analysis was done by Chi-square, and Spearman’s correlation using SPSS statistical software at a significant level of (P < 0.05).

**Results:**

The results showed unfavorable PAL, eating behavior, QoL, GH, and mood states in the total population. Low PAL was observed in 69.96% of the men and 75.99% of the women; only 3.60% of the men and 0.77% of the women had a high PAL. There was a significantly positive relationship between low PAL and the incidence of COVID-19 both in men and women (P = 0.801; r = 0.001; and P = 0.682; r = 0.011), respectively; While a significant negative relationship was observed between the moderate and high PAL and the incidence of COVID-19 in men (P = 0.011; r=-0.682 and P = 0.027, r=-0.589), and women (P = 0.001; r=-0.796 and P = 0.018, r=-0.623). No significant relationships were observed between PAL and eating behavior (men: P = 0.086; r = 0.256 and women: P = 0.365, r=-0.121); While, the results show significant positive relationships between PAL with QoL in men (P = 0.012; r = 0.623) and women (P = = 0.001; r = 0.837). based on the results, significant negative relationships between PAL with GH and mood state scores were observed in both men (P = 0.001; r=-0.837 and P = 0.001, r=-0.786) and women (P = 0.010; r=-0.652 and P = 0.001, r=-0.745), respectively.

**Conclusions:**

The Iraqi adult population showed low PAL, GH, QoL, and mood state during COVID-19 which might be due to the confinement. Also, the significant relationships between low PAL with GH, and mood state recommends physical activity as a valuable health optimizing factor during the COVID-19 pandemic.

## Introduction

Coronavirus disease 2019 (COVID-19) is an infectious disease induced by newly found Severe Acute Respiratory Syndrome Coronavirus 2 (SARS-CoV-2), reported first in Wuhan [1]. To this end, health authorities imposed confinement regulations to reduce the spreading of COVID-19, including social distancing, wearing face masks, locking down cultural and sports events, staying in quarantine if the infection is doubted, and isolation if the infection is approved [1]. Home confinement harmed eating behavior, and caused strict restrictions on regular exercise training or physical activities, especially in the population with underlying diseases [2, 3]. Thus, remained long-term consequences on General Health (GH), and Quality of Life (QoL) are not unexpected [2, 4, 5]. Recent literature investigated Physical Activity Level (PAL) during confinement; However, there were inconsistent results on the association between physical activity patterns and confinement conditions [6, 7]. Lesser et al. (2020) showed that PAL decreased among 40.5% while increased among 33% of modestly active adults, also that 22.4% of active adults got less active whereas 40.3% of active people got more active during the lockdown [8]. Also, Phillipou et al. (2020) investigated eating and exercise behaviors during the COVID-19 pandemic in Australia reporting both increased restricting and binge eating behaviors and less exercise relative to before the pandemic in the general population [9]. A similar study in Italy compared the data collected three weeks to the social lockdown with the confinement period in 41 obese children and adolescents reporting relatively less exercise and increased consumption of ‘unhealthy’ junk foods [10]. While, Constandt et al. observed that adults with low PAL before the confinement reported exercising more while those with high PAL reported exercising less with increased sitting time during the confinement [11]. Given this package of data, it appeared plausible that the effect of the COVID-19 crisis on eating behavior and physical activity is indefinite but substantial [12, 13]. Additionally, physical activity is an essential key factor affecting mood state, GH, and QoL in both healthy and clinical samples [14–16]. While some study observed neither increased nor decreased PAL and showed no alteration in GH and mood state among Iranian adults attending team sports activities during the COVID-19 pandemic [17]. Other studies approved that COVID-19 outbreak deteriorated the mood state and reduced GH and QoL [13, 18]. Also, previous studies investigating the psychological distress among adults reported poor mood state, particularly in women [19, 20]. To date, no research has explored the alteration of PAL, eating behavior, QoL, GH, and mood states during the COVID-19 pandemic in the Iraqi population. To counterbalance the lack of such research, we aimed to investigate the PAL, eating behavior, QoL, GH, and mood states during COVID-19 confinement in a large sample of Iraqi adults.

## Materials and methods

### Study design and population

This applied research studied the men and women of Halabja, Iraq using the available sampling method from August to December 2021; a period in which Iraq faced a high infection rate with an average of 83098 new cases per week and 2465545 total cases up to now [21]. For this purpose, the statistical population of this cross-sectional-descriptive study consisted of all Iraqi adults in Halabja city aged 18–70 years. According to Chirico’ study [22], and Khani et al [23] the sample size was estimated 4000 Iraqi citizens using a convenience sampling approach. The questionnaire was sent to available samples online using Telegram, WhatsApp, and other social media applications, of which 3927 individuals filled the questionnaire. After the final investigations and excluding the incomplete answers, 3738 individuals (1914 men and 1824 women) were considered for further analyses.

Participants were informed about the aims of the study, the confidential data handling, and the ethical approval of the study at the beginning of the questionnaires. Next, to provide the informed consent online, participants clicked the box of the agreement to participate as volunteers. The study was performed following the seventh and current revision (World Medical Association, 2013) of the Declaration of Helsinki. Also, all educated participants and the legal guardian of illiterate participants were asked to complete the written informed consent at the beginning of the study.

### Measures

#### Sociodemographic Information

Participants reported their age (years), sex (women, men), bodyweight (BW), height, marital status (single, married), educational level (illiterate, undergraduate, and college), occupational tatus (employed, unemployed, and student), smoking, and Hospitalization in the last.

### Physical activity levels

To determine the PAL Short Form of International Physical Activity Questionnaire (SF-IPAQ) was utilized in this study. IPAQ contains 7 questions classifying all types of physical activity lasting longer than 10 min within the last 7 days including leisure time, domestic and gardening (yard) activities, work-related and transport-related activities [24, 25] into three categories: walking, moderate and vigorous [26]. The Walking category included all types of walking; Moderate category included carrying light loads, cycling, swimming, volleyball, and all other activities that cause small increases in breathing and/or heart rate; Vigorous category covers all physical activities that cause large increases in breathing and/or heart rates such as carrying or lifting heavy loads, digging, running, football or construction work. Frequency (days per week) and duration (time per day) were recorded. The Metabolic Equivalent (MET) was calculated in kcal/kg/hour for each activity. Eight METs were considered for vigorous activities, 4 METs for moderate, and 3.3 METs for walking. Then, the score for each activity was calculated by multiplying its METs *frequency (days per week) * duration (time per day in minutes). The total PAL was calculated by adding the Walking, Moderate, and Vigorous activities MET scores. High PAL was defined as scores > 1500 MET, moderate PAL as scores between 600 and 1500 MET, and low PAL as any scores < 600 MET [24, 25].

### Eating behavior

In this study, the 18-item Three-Factor Eating Questionnaire (TFEQ-R18) was used that measures 3 different aspects of eating behavior: uncontrolled eating (UE; intended to eat more than usual due to a loss of control over intake combined with subjective feelings of hunger, 9 questions), conscious restriction (CR; restriction of food intake to control body weight or to promote weight loss, 6 questions), and emotional eating (EE; inability to resist emotional cues, 3 questions). It has been validated that the instrument is valid in both obese and non-obese individuals [27]. The responses were coded on a 4-point scale (1 = definitely true, 2 = mostly true, 3 = mostly false, 4 = definitely false) with higher values indicating more of the behavior. The raw scale scores are standardized to a 0–100 scale using the following formula:

Standardized score = [(raw score-lowest possible raw score)/possible raw score range] × 100.

### General health

To assess and identify health problems within the past two weeks we used a 28-item GH questionnaire (GHQ-28; including somatic symptoms, anxiety and insomnia, social dysfunction, and depression subscales, each with seven questions) which was developed and psychometrically evaluated by Goldberg and Homan, respectively [28]. The responses were scored with 4 points of 0, 1, 2, and 3 thus the scores for each subscale could be between 0 and 21 and the total GHQ-28 score between 0 and 84 in which higher scores represent lower GH and any scores above 23 were considered as impaired GH [28]. The validity of the Persian version of GHQ-28 for assessing the GH in the Iranian population was reported by Taghavi previously[29].

### Mood state

The Short Form of the Profile of Mood States (POMS-SF) is a reliable and valid measure of subjective mood states that has been used extensively in a wide variety of studies [30]. This questionnaire has 37 questions in six subscales including tension or anxiety, anger or hostility, vigor or activity, fatigue or inertia, depression or dejection, and confusion or bewilderment. Participants respond on a five-point, Likert-type scale ranging from 0 (not at all) to 4 (extremely). The total mood states score was computed by adding the five negative subscale scores (tension-anxiety, depression, anger-hostility, vigor, fatigue, and confusion) and subtracting the vigor score. Higher scores for the total mood state score indicate a greater degree of mood disturbance. The reliability of the Persian of POMS-SF using Cronbach’s alpha coefficient was acceptable in the range of 0.74 to 0.88.

### Quality of life

The QoL was assessed using the SF-12 was developed as a shorter alternative to the SF-36, applicable to large-scale health surveys where the application of the more extended instrument would be too time-consuming or costly. The SF-12 has 12 questions which are grouped into eight domains or two summary components of the physical component and mental component scales of physical functioning, role limitations due to physical health, bodily pain, GH perception, social functioning, role limitations due to emotional problems, vitality, and mental health. The final score of the SF-12 ranged from 0–to 100, with a higher score indicating a better QoL. Cronbach’s alpha coefficient was 0.88, indicating a high internal consistency. The SF 12 was adapted and validated to Persian conditions by Rohani et al. [31]. Concerning criterion validity, correlations between the subscales of the SF-12 were 0.63–0.78.

### Statistical analysis

All statistical analyses were performed using the SPSS statistical software (version 21; SPSS Inc., Chicago, IL, USA) at a significant level of P < 0.05. The Kolmogorov–Smirnov test was used to evaluate the normality of distribution. The descriptive (mean, standard deviation, and percent) and deductive (Chi-square and Spearman’s correlation) statistic methods were used for analyzing the data.

## Results

Table 1 shows the Mean ± SD of anthropometric characteristics and physiologic variables among the participants. Based on these results, the subjects were overweight (men: 28.50 ± 7.82; women: 29.51 ± 8.29) and had an insufficient PAL (men: 571.75 ± 608.56; women: 539.94 ± 451.38).

**Table 1:** Anthropometric characteristics of participants

Variable	Mean ± SD		
	**Total**	**Men (n = 1914)**	**Women (n = 1824)**
Age (Years)	44.96 ± 14.26	45.42 ± 14.18	44.47 ± 14.34
BW (kg)	79.20 ± 20.18	84.09 ± 20.74	74.08 ± 18.23
Height (cm)	166.20 ± 12.15	172.65 ± 10.47	159.42 ± 9.89
BMI (kg/m^2^)	28.99 ± 18.07	28.50 ± 7.82	29.51 ± 8.29
PAL (MET)	556.23 ± 537.80	571.75 ± 608.56	539.94 ± 451.38

**BW;** Bodyweight; **BMI**: Body Mass Index; **PAL**: Physical Activity Level

The results showed that 36.90% of the participants in the present study were in the older adults age (36–55) category and also 72.45% of the participants in the present study were married (Table 2), significant differences were observed between men and women in marital status. In concern with educational level, only 31.30% of the participants had a college education. According to the results, significant differences were observed between men and women in the educational level. In addition, 44.23% of the participants in the present study were unemployed and there were significant differences in the rate of unemployment between men and women (men: 35.60%; women: 53.2%). Also, only 73.92% of the participants in the study were cigarette smokers; The highest frequency of smoking was observed in men (35%). According to the results of Table 2, 41.20% of men and 48% of women experienced hospitalization in the last year which showed a significant difference (Table 2).

**Table 2:** Demographic status of participants in the total population and different sex categories

Variable	Total (n = 3738)	Men (n = 1914)	Women (n = 1824)	Statistics
Age group (years)				
18–35	1093 (29.10%)	524 (27.40%)	569 (31.20%)	0.221
36–55	1387 (36.90%)	717 (37.50%)	670 (36.70%)	
55 <	1258 (33.50%)	673 (35.20%)	585 (32.10%)	
Marital status				0.001*
Single	1030 (27.55%)	574 (30%)	456 (25%)	
Married	2708 (72.45%)	1340 (70%)	1368 (75%)	
Educational level				0.001*
Illiterate	734 (19.63%)	328 (17.10%)	406 (22.30%)	
Undergraduate	1834 (49.07%)	987 (51.60%)	847 (46.40%)	
College	1170 (31.30%)	599 (31.30%)	571 (31.30%)	
Occupational status				
Employed	1277 (34.16%)	848 (44.30%)	429 (23.50%)	0.001*
Student	808 (21.61%)	384 (20.10%)	424 (23.20%)	
Unemployed	1653 (44.23%)	682 (35.60%)	971 (53.20%)	
Smoking				
Yes	975 (26.08%)	669 (35%)	306 (16.80%)	0.001*
No	2763 (73.92%)	1245 (65%)	1518 (83.20%)	
Hospitalization in the last year				
Yes	1665 (44.54%)	789 (41.20%)	876 (48%)	0.001*
No	2073 (55.46%)	1125 (58.80%)	948 (52%)	

Data analysis was done by the χ2 test

*: Significantly different, comparing men and women

Table 3 presents the frequency and percentage of COVID-19 symptoms in 3 categories (less common, more common, and serious symptoms). Based on the results, almost all subjects reported having all of the symptoms of the first (less-common symptoms; 39.98% of men and 36.57% of women) and second categories (common symptoms; 39.65% of men and 42.49% of women). while difficulty breathing was the most reported symptom in the third category (33.03% of men and 29.17% of women). There was significant difference between men and women in the COVID-19 symptoms.

**Table 3:** The frequency and percentage of COVID-19 symptoms among men and women

Categories	Total (n = 3738)	Men (n = 1914)	Women (n = 1824)	P-Value
**Less common Symptoms**				0.001*
**Soreness and pain**	512 (13.70%)	226 (11.81%)	286 (15.68%)	
**Sore throat**	351 (9.39%)	189 (9.87%)	162 (8.88%)	
**Diarrhea and Vomiting**	269 (7.20%)	148 (7.73%)	121 (6.63%)	
**Inflation**	223 (5.96%)	125 (6.53%)	98 (5.37%)	
**Headache**	525 (14.04%)	256 (13.37%)	269 (14.75%)	
**Loss of smell or taste**	226 (6.05%)	129 (6.74%)	97 (5.32%)	
**Pimples or paleness of the fingers and toes**	200 (5.35%)	76 (3.97%)	124 (6.80%)	
**All**	1432 (38.31%)	765 (39.98%)	667 (36.57%)	
**More Common Symptoms**				0.001*
**Fever**	760 (20.33%)	368 (19.23%)	392 (21.50%)	
**Dry cough**	587 (15.70%)	289 (15.10%)	298 (16.34%)	
**Fatigue**	857 (22.93%)	498 (26.02%)	359 (19.67%)	
**All**	1534 (41.04%)	759 (39.65%)	775 (42.49%)	
Serious Symptoms				0.001*
**Difficulty breathing**	1164 (31.14%)	632 (33.03%)	532 (29.17%)	
**Chest pain or pressure**	653 (17.47%)	368 (19.23%)	285 (15.62%)	
**Losing the ability to move or speak**	393 (10.51%)	228 (11.91%)	165 (9.05%)	
**All**	801 (21.43%)	314 (16.40%)	487 (26.70%)	
**None**	727 (19.45%)	372 (19.43%)	355 (19.46%)	

Data analysis was done by the χ2 test

*: Significantly different, comparing men and women

Also, a significantly positive relationship between low PAL and the incidence of COVID-19 both in men and women (P = 0.801; r = 0.001; and P = 0.682; r = 0.011), respectively; While a significant negative relationship was observed between the moderate and high PAL with the incidence of COVID-19 in men (P = 0.011; r=-0.682 and P = 0.027, r=-0.589), and women (P = 0.001; r=-0.796 and P = 0.018, r=-0.623) (Table 4).

**Table 4:** The relationship between the PAL and the incidence of COVID-19

	PALs					
Incidence of COVID-19	Men			Women		
	Low	Moderate	High	Low	Moderate	High
	r = 0.801 P = 0.001 ^*^	r=-0.682 P = 0.011 ^*^	r=-0.589 P = 0.027 ^*^	r = 0.726 P = 0.002 ^*^	r=-0.796 P = 0.001 ^*^	r=-0.623 P = 0.018 ^*^

Data analysis was done by the Spearman’s correlation test

*: Significant relationship with the incidence of COVID-19

The results showed unfavorable, PAL, eating behavior, QoL, GH, and mood states in the total population. Low PAL was observed in 69.96% of the men and 75.99% of the women; only 3.60% of the men and 0.77% of the women had a high PAL. The results showed significant differences between different genders in the low (P = 0.032), moderate (P = 0.041), and high (P = 0.045) PAL. Also, uncontrolled eating behavior was observed in 50.57% of the men and 47.37% of the women while 38.09% of the men and 41.45% of the women were emotional eaters; and 11.34% of the men and 11.18% of the women had conscious restraints. There were no significant differences in the eating behavior variables between men and women. The results indicate poor GH, QoL, and mood state in all participants. However, there were no significant differences in the GH, QoL, and mood state between men and women; but both genders showed low GH, QoL, and mood state (Table 5).

**Table 5:** PAL, Eating behavior, QoL, GH, and mood states

Variable	Total N = 3738	Men (n = 1914)	Women (n = 1824)	Statistics
**PAL (%)**				
Low level	2725 (72.90%)	1339 (69.96%)	1386 (75.99%)	P = 0.032*
Moderate level	930 (24.88%)	506 (26.44%)	424 (23.24%)	P = 0.041*
High level	83 (2.22%)	69 (3.60%)	14 (0.77%)	P = 0.045*
**Eating behavior (%)**				
Uncontrolled eaters (UE)	1832 (49.01%)	968 (50.57%)	864 (47.37%)	P = 0.075
Conscious restraints (CR)	421 (11.26%)	217 (11.34%)	204 (11.18%)	P = 0.691
Emotional eaters (EE)	1485 (39.73%)	729 (38.09%)	756 (41.45%)	P = 0.102
**QoL**				
GH	25.38 ± 1.75	25.92 ± 1.18	24.85 ± 2.32	P = 0.069
Physical Functioning	19.41 ± 1.87	19.56 ± 2.01	19.26 ± 1.73	P = 0.476
Role Physical	25.84 ± 2.27	26.64 ± 3.07	25.04 ± 1.47	P = 0.053
Body Pain	25.71 ± 2.47	25.65 ± 1.86	25.78 ± 3.09	P = 0.501
Vitality	22.94 ± 2.34	23.56 ± 2.9	22.33 ± 1.78	P = 0.059
Social Functioning	22.69 ± 2.26	22.12 ± 1.15	23.26 ± 3.38	P = 0.066
Role Emotional	19.07 ± 2.02	19.30 ± 1.96	18.85 ± 2.08	P = 0.381
Mental health	20.99 ± 1.72	21.12 ± 2.18	20.87 ± 1.26	P = 0.547
Overall QoL	23.01 ± 2.09	23.48 ± 2.04	22.53 ± 2.14	P = 0.236
**GH questioners-28 scores**				
Anxiety and insomnia	13.99 ± 2.07	13.45 ± 2.03	14.53 ± 2.11	P = 0.085
Somatic symptoms	12.10 ± 2.02	11.56 ± 2.42	12.65 ± 1.62	P = 0.082
Social impairment	16.77 ± 2.70	16.21 ± 2.37	17.34 ± 3.04	P = 0.061
Depression	7.71 ± 0.32	7.59 ± 0.27	7.83 ± 0.38	P = 0.460
Total score	12.64 ± 1.78	12.20 ± 1.77	13.09 ± 1.79	P = 0.145
**Mood states scores**				
Confusion	7.67 ± 0.20	7.56 ± 0.22	7.78 ± 0.19	P = 0.410
Anger	8.30 ± 0.25	8.15 ± 0.34	8.46 ± 0.17	P = 0.274
Depression	9.52 ± 0.36	9.23 ± 0.32	9.82 ± 0.41	P = 0.189
Vigor	6.21 ± 0.18	6.12 ± 0.12	6.30 ± 0.24	P = 0.393
Fatigue	9.24 ± 0.27	9.06 ± 0.43	9.43 ± 0.11	P = 0.240
tension	8.67 ± 0.19	8.62 ± 0.14	8.73 ± 0.25	P = 0.691
Total score	8.27 ± 0.24	8.12 ± 0.26	8.42 ± 0.23	P = 0.295

Data analysis was done by the χ2 test

*: Significantly different, comparing men and women

The results of show significant negative relationships between PAL with BMI in both men and women (P = 0.003; r=-0.690; and P = 0.093; r=-0.649), respectively (Table 6). While no significant relationships were observed between PAL and eating behavior (men: P = 0.086; r = 0.256 and women: P = 0.365, r = 0.121). Also, the result shows significant positive relationships between PAL with QoL in men (P = 0.012; r = 0.623) and women (P = = 0.001; r = 0.837). While significant negative relationships between PAL with GH and mood state scores were observed in both men (P = 0.001; r=-0.837 and P = 0.001, r=-0.786) and women (P = 0.010; r=-0.652 and P = 0.001, r=-0.745), respectively (Table 6). It should be mentioned that higher scores in GH and mood state indicate lower GH and higher mood disturbance.

**Table 6:** Relationship between PAL with BMI, Eating behavior, QoL, GH, and mood state among men and women

Variables	PAL			
	**Men**		**Women**	
	**P**	**β**	**P**	**β**
**BMI**	P = 0.003	r=-0.690	P = 0.093	r=-0.649
**Eating behavior**	P = 0.086	r = 0.256	P = 0.365	r = 0.121
**QoL**	P = = 0.012	r = 0.623	P = = 0.001	r = 0.837
**GH**	P = = 0.001	r=-0.837	P = = 0.001	r=-0.786
**Mood state**	P = 0.010	r=-0.652	P = = 0.001	r=-0.745

Data analysis was done by the Spearman’s correlation test

*: Significant relationship with the incidence of COVID-19

The results show significant negative relationships between COVID-19 incidence with PAL (P = 0.001; r=-0.832; and P = 0.021; r=-0.520), QoL (P = 0.023; r=-0.541; and P = 0.001; r=-0.802) in both men and women, respectively (Table 7). While no significant relationships were observed between COVID-19 incidence with eating behavior (men: P = 0.263; r = 0.142 and women: P = 0.295, r = 0.216). Also, significant positive relationships between COVID-19 incidence with GH and mood state scores were observed in both men (P = 0.001; r = 0.769 and P = 0.018, r = 0.591) and women (P = 0.001; r = 0.794 and P = 0.001, r = 0.841), respectively (Table 7); considering the higher scores indicating lower GH and mood state.

**Table 7:** Relationship between COVID-19 incidence with PAL, Eating behavior, QoL, GH, and mood state among men and women

Variables	COVID-19 incidence			
	**Men**		**Women**	
	**P**	**β**	**P**	**β**
**PAL**	P = 0.001	r=-0.832	P = 0.021	r=-0.520
**Eating behavior**	P = 0.263	r = 0.142	P = 0.295	r = 0.216
**QoL**	P = = 0.023	r=-0.541	P = = 0.001	r = 0.802
**GH**	P = = 0.001	r = 0.769	P = = 0.001	r = 0.794
**Mood state**	P = 0.018	r = 0.591	P = = 0.001	r = 0.841

Data analysis was done by the Spearman’s correlation test

*: Significant relationship with the incidence of COVID-19

## Discussion

This research aimed to analyze the effect of COVID-19 confinement on PAL, eating behavior, GH, QoL, and mood states in a large sample of Iraqi adults. The study showed that Iraqi adults significantly decreased PAL, QoL, GH, and mood states while increased eating disorders during the confinement period. Also, subjects with lower PALs, both men, and women, showed poorer eating behavior, QoL, GH, and mood state.

Regarding the health impact of COVID-19 confinement, poorer mood state and lower PAL, resulting from the extended period of confinement perception, fear of getting infected, boredom, and frustration were described [32]. In line with our results Azmodeh et al. (2021) showed inadequate PAL, low QoL, and GH in men and women recovering from COVID-19; also showing sex differences between men and women that were not observed in the present study [6]. These findings are in line with Amini et al. (2020) investigating Iranian adults that showed low physical activity in the majority of the participants during COVID-19 [33]. In contrast, Aghababa et al. (2020) observed decreased physical activity intensity but increased physical activity frequency among Iranian adults during the confinement compared with before stating no evidence of systematic change in physical activity patterns and related mood states [17].

Based on the results of the studies, COVID-19 confinement has induced extensive lifestyle changes in different populations that are predicted to continue for a long time. Among these dramatic changes was the disruption of eating habits and eating patterns. Although the results indicated poor eating behavior between men and women, the results of the present study indicate no significant relationship between PAL and eating behavior. Another study that investigated the impact of COVID-19 on eating behavior among the Italian population reported higher adherence to a healthy diet in the age group of 18–30 years compared to the younger and the elderly population [34]. Also, a recent study in Saudi Arabia observed that the lockdown significantly impacted the physical activity and dietary behaviors of several citizens and residents in an unhealthy way [35]. Also, a previous study that assessed the effect of the lockdown on eating habits in the United Arab Emirates (UAE) reported a 31% weight gain while an increased rate of unhealthy eating behavior [36]. The eating behavior alterations might be due to boredom, anxiety, low motivation to have a healthy diet, reduced goods availability, and limited food access considering store opening restrictions [37]. These changes in eating behavior together with low PAL might lead to negative effects on GH, QoL, and mood state.

Interestingly, our results show significant negative relationships between PAL with GH, and mood state; considering the higher scores showing poorer results in the mentioned variables. In line with our results, studies indicate adequate PAL improves the GH [38, 39] and mood state [38, 40]. Overall, maintaining adequate PAL in men and women might benefit GH and mood states. Although the main mechanism of exercise improving GH is not fully elucidated, increased PAL and exercise help improve anxiety, confusion, anger, depression, insomnia, and social dysfunction [41], thus optimizing GH and mood state in both men and women probably via increasing serotonin, stress hormones, and endorphins in the brain [42]. In addition, positive correlations were found between the PAL and QoL among both men and women. In other words, increased QoL was observed with increased PAL. Similar results were obtained in a previous study regarding a positive and significant relationship among patients with COVID-19 [43]. Based on the results of other studies physical activity can promote QoL through its consequent physical and psychological benefits including reduced risk of illness, improved immune response, higher mental stimulation, increased social interactions, and improved self-esteem [44, 45]. Focusing on gender-based differences, in this study men tend to show better results in the measured variables however these differences were not significant between men and women.

### Limitation

The acceptance rate to contribute in this study varied among different families and some refused to participate in this study. Also, there were areas with no internet network, so using Telegram, WhatsApp, and other social media applications were not possible in the people of that areas. Although we had a quiet large sample size in this study, it is more considerate not to generalize this information to the whole society considering the limitations that were stated above.

## Conclusion

In this study, we have provided for the first time data on the Iraqi population. In general, the findings of the present study showed that the Iraqi adult population showed low PAL, GH, QoL, and mood state during COVID-19 confinement that might be due to the increased stress, and anxiety caused by the COVID-19 pandemic. Also, the significant relationships between low PAL with GH, and mood state recommends physical activity as a valuable health optimizing factor in the COVID-19 pandemic.

## Data Availability

The datasets generated and analyzed during the current study are not publicly available due to ongoing data analysis but are available from the corresponding author on reasonable request.
